# ‘It looks like a breadbox’: a pilot study investigating implementation of the Pepi-Pod® program with Aboriginal families in metropolitan South Australia

**DOI:** 10.1017/S1463423621000293

**Published:** 2021-06-10

**Authors:** Julian Grant, Nina Sivertsen, Janiene Deverix, Alice Steeb

**Affiliations:** 1 School of Nursing, Midwifery and Indigenous Health, Charles Sturt University, Bathurst, NSW 2795, Australia; 2 Flinders University, Bedford Park, SA, Australia; 3 Aboriginal Health Strategic Operations, Aboriginal Health Strategic Operations, Child and Family Health Services, SA Health, Adelaide, SA, Australia; 4 Child and Family Health Services, SA Health, Adelaide, SA, Australia

**Keywords:** culture, child health, Indigenous health services, maternal health services, postnatal care, perinatal care

## Abstract

**Aims:**

To collaboratively explore the cultural acceptance of the Pepi-Pod® program as an alternate safe sleep space and to explore the process of implementing the Pepi-Pod® program in a mainstream health service for Aboriginal families living in urban South Australia.

**Background:**

Aboriginal and Torres Strait Islander infants continue to die from sudden infant death syndrome (SIDS) and sudden unexpected death in infancy (SUDI) at rates three to four times greater than other infants born in Australia despite Council of Australian Governments commitment to halve the gap in the Indigenous infant mortality rate by 2018. The Pepi-Pod® program is evidenced in New Zealand and Queensland to provide a culturally appropriate safe sleep alternative that contributes to the reduction of SIDS and SUDI. We have no evidence of acceptability or feasibility when offered through mainstream services in metropolitan South Australia.

**Methods:**

With a focus on decolonizing the research process through a two-way process for mutual learning between Aboriginal and non-Aboriginal team members and community, a novel qualitative design was employed including photo elicited yarning sessions (*n* = 7), focus groups (*n* = 2), and field notes (*n* = 15).

**Results:**

Four themes emerged: ‘*you don’t have to worry’*; ‘a way of sharing knowledge’; ‘*it looks like a bread box?’* and ‘need for consistent safe sleep messages’. The findings suggest that participants believe the Pepi-Pod® program may enrich Aboriginal families’ lives evoking feelings of comfort and safety; however, the design could be improved to make them more culturally appropriate. There was confusion around safe sleep processes and education with a call for streamlining safe sleep messaging.

## Introduction

Aboriginal and Torres Strait Islander infants die from sudden infant death syndrome (SIDS) and sudden unexpected death in infancy (SUDI) at rates 3–4 times greater than other infants born in Australia (Australian Health Ministers’ Advisory Council (AHMAC), 2017). Despite a significant reduction in SIDS and SUDI for Aboriginal infants in the past 20 years (Australian Health Ministers’ Advisory Council (AHMAC), 2017) the difference remains unacceptable, given that the Council of Australian Governments (Council of Australian Governments (COAG), 2018) committed in 2007 to halving the gap in the Indigenous infant mortality rate (IMR) by 2018.

In South Australia (SA), there were 29 deaths of Aboriginal and Torres Strait Islander infants between 2005 and 2013 where unsafe sleeping environments were noted (Child Death and Serious Injury Review Committee 2014). All Aboriginal families birth in mainstream health services in SA including the state-wide Women and Children’s Health Network (WCHN). As part of WCHN, the Child and Family Health Service (CaFHS) provides well child health care to all children from birth until 6 years. Midwives and child and family health nurses (CaFHNs) hold primary responsibility for education and support around safe sleeping. Late in 2014 following an inquest into the death of a 3-month-old infant, the Coroner made recommendations impacting on infant sleeping practices promulgated through CaFHS (Coroner’s Court of SA 2014). The Coroner observed that CaFHS nurses inadequately recorded their observations of the infant sleeping arrangements and that there was inadequate notation of safe sleeping advice (Coroner’s Court of SA 2014). The coroner recommended that:

*As part of any CaFHS home visit assessment of an infant’s circumstances, CaFHS nurses and other workers should thoroughly investigate and document the sleeping environment of an infant within the infant’s home and that such investigation and documentation should take place on each and every home visit. Where possible, photographic evidence should be obtained in relation to the sleeping environment. Refusals on the part of parents to allow the sleeping environment to be viewed should be documented and should in the normal course generate concern. Naturally, robust efforts should be made to correct any infant sleeping practice that is intrinsically dangerous and/or presents risk of sudden infant death while sleeping (* *Coroner’s Court of SA 2014)*.

Additional recommendations included documentation of education and advice on sleeping environments and practices (Coroner’s Court of SA 2014). At the time, SA Health held a risk elimination strategy for safe sleeping (Government of South Australia, 2011) where families were advised to place their baby in a cot next to the bed. This provided no option for health professionals to offer harm minimisation strategies or culturally safe options for safe sleeping. Such professional tensions can result in culturally unsafe and racist practice (Grant and Guerin, 2018).

The organizational response required completion of a ‘*Sleeping Baby Safely form*’. Aboriginal Cultural Consultants (ACCs) and CaFHNs identified that many Aboriginal families struggled to provide safe sleeping spaces for their infants with limited access to appropriate bedding and that the cultural practice of sleeping the infant in the parent/caregiver’s bed was common. Completion of the form was perceived as culturally insensitive and argued to result in disengagement of families who identified as Aboriginal.

To address these concerns, a team of ACCs and CaFHNs formed a working group to identify evidence informed, alternative safe sleeping options that could be offered to Aboriginal and Torres Strait Islander families using mainstream services.

This paper reports on the findings of a pilot study trialling use of the Pepi-Pod® program through mainstream health services with urban Aboriginal families living in South Australia. Using a novel approach, the research aimed to identify if the program and alternate sleeping space was perceived as culturally safe and to explore the process of implementing the Pepi-Pod® program in a mainstream health service.

## Background

Disparities in health outcomes between Aboriginal and Torres Strait Islander and other Australians are well documented (Shepherd et al., 2012). Likewise, these disparities exist in the IMR and in SUDI and SIDS. While the Indigenous IMR declined significantly between 1998 and 2015 (13.5–6.3 per 1000 live births) and the gap between Indigenous and non-Indigenous infant mortality reduced by 84%, the Indigenous IMR was still 1.9 times the non-Indigenous rate between 2011 and 2015 (ibid). This is a significant clinical, cultural, and sociological issue.

SUDI pertains to babies who die under 1 year of age, usually during sleep, and can be explained or unexplained (Fleming et al., 2015). SIDS is a subset SUDI where the fatal episode is apparently during sleep and is unexplained (ibid). The triple risk model (Filiano and Kinney, 1994) is a widely accepted framework for understanding the intrinsic and extrinsic factors contributing to SIDS. It brings together evidence on infant critical development periods, underlying predisposition and environmental factors, contending that together they result in acute vulnerability (ibid).

Bed-sharing is an environmental factor that has been the focus of preventative action since the 1990s. It is defined as ‘*bringing baby onto a sleep surface when co-sleeping is possible, whether intended or not*’(United Nations International Children’s Emergency Foundation (UNICEF), 2013) and generally refers to an infant and adult sleeping on the same surface where the infant may share the sleep surface all night or for a short period (Baddock et al., 2018). ‘*Co-sleeping’* is a term often used interchangeably with bed-sharing; however, it can include room-sharing which is evidenced to reduce the risk if SUDI by up to 50% (Carpenter et al., 2004). For the purposes of this paper, we refer specifically to adults ‘*sharing the same sleep surface with an infant*’ (SA Health, 2018) with reference to sleeping together on any surface including a bed, couch, or chair.

Bed-sharing is a common practice in many Western societies including Australia, New Zealand, the United Kingdom, and USA (Ball and Volpe, 2013). It is argued to have many benefits including increased success and duration of breastfeeding (Huang et al., 2013) and enhanced maternal responsiveness and attachment (Cunningham, 2015). Sharing a sleep surface is a valued infant care practice in Australia (Australian College of Midwives’ 2016), and a valued cultural norm in many Aboriginal and Torres Strait Islander communities (Desmosthesous C and T, 2011) with 68%–77% of Aboriginal families in northern Queensland and metropolitan Western Australia sharing a sleep surface with their babies (Panaretto et al., 2002, Eades and Read, 1999).

Infant deaths are associated with sharing a sleep surface where there are additional complex and hazardous circumstances (Mitchell, 2015). Widening socioeconomic inequities are increasingly recognized as contributing to the complexity of the problem, including the capacity to provide a safe sleep environment (Shipstone et al., 2017, Freemantle et al., 2006, Knight et al., 2013). While international data suggest that Indigenous infants globally are exposed to higher risks of SIDS and SUDI (Hauck and Tanabe, 2008), there is limited national Australian data pertaining specifically to risks factors.

### The collaboration

The initial working group was extended to operationalize the project. It comprised ACCs, CaFHNs, representatives from SA Aboriginal Health Services and the Aboriginal Family Birthing Program, the SA Health Public Health Service, Kidsafe SA, Aboriginal Health Council of South Australia, SIDS and Kids SA, and researchers from Flinders University. Many portable safe sleep surfaces were considered, for example, from Finland, United Kingdom, and North America; however, the empirical evidence related to their efficacy was and remains poorly substantiated by research data (Blair et al., 2018). The Pepi-Pod® program was identified as an evidence informed option with potential to meet the needs of parents, community, and industry. At the time, it had been reported to reduce the risks of SIDs in New Zealand and in Queensland, Australia (Cowan, 2015, Young et al., 2015).

The Pepi-Pod® program comprises three interlinked components; a portable safe sleep space, health education delivered by a known health care professional, and family commitment (Cowan, 2015). It enables infants to sleep on their own sleep surface on or near an adult bed, close to their carer. As such it complied with the risk elimination approach of the SA Safe Infant Sleep Standards (Government of South Australia, 2011).

While Queensland data indicate that Aboriginal families accepted the Pepi-Pod® program, there is no evidence to suggest that cultural acceptance can be translated to another group of Aboriginal or Torres Strait Islander peoples. Further, the Queensland trial incorporated both Aboriginal Community Controlled and mainstream services, incorporating both antenatal and postnatal services (Young et al., 2018). There was no comparative analysis regarding acceptance between services or sites. While best practice for Aboriginal and Torres Strait Islander health delivery is with Aboriginal Community Controlled services (Campbell et al., 2018), mainstream services hold a mandate to provide care that is experienced as culturally safe (The Wardliparingga Aboriginal Research Unit of the South Australian Health and Medical Research Institute, 2017). The aim was to collaboratively explore both the cultural acceptance of the Pepi-Pod® program as an alternate safe sleep space and the process of implementing the Pepi-Pod® program in a mainstream health service for Aboriginal families living in urban South Australia. A further agreed priority was to facilitate the research to be experienced as culturally safe as possible for participating Aboriginal families.

## Methods

The research was underpinned by the principles of health equity with a focus on decolonizing the research process (Laycock *et al.*, 2011). A two-way process was used for mutual learning between Aboriginal and non-Aboriginal team members and community (Durie, 2005). This was enacted through ‘*two-eyed seeing*’ where Aboriginal and Western world views of health and illness come together (Sivertsen et al., 2020) for the benefit of all. This was particularly important as the research was conducted though mainstream health services where unequal relations of power have historically compromised Aboriginal ways of knowing and being (DiGiacomo *et al.* 2019).

Following receipt of grant funding, the original working group was renamed as the Research Advisory Group (RAG). The project initiator identified as Aboriginal in addition to a further three original members. They indicated that given the limited time frame of the funding that they would be best to use their existing personal and professional networks to seek project management advice from Aboriginal community members, rather than establishing a project-specific Aboriginal Advisory Group. The RAG met monthly and committed to individual phone and email communications to ensure non-Aboriginal researchers could seek advice on the cultural appropriateness of activities at any time, and that all members could work together to operationalize the research. The RAG also developed a fluidity whereby relevant industry and/or Aboriginal community members were invited to address the team on specific issues.

### Study design

**Figure 1.**
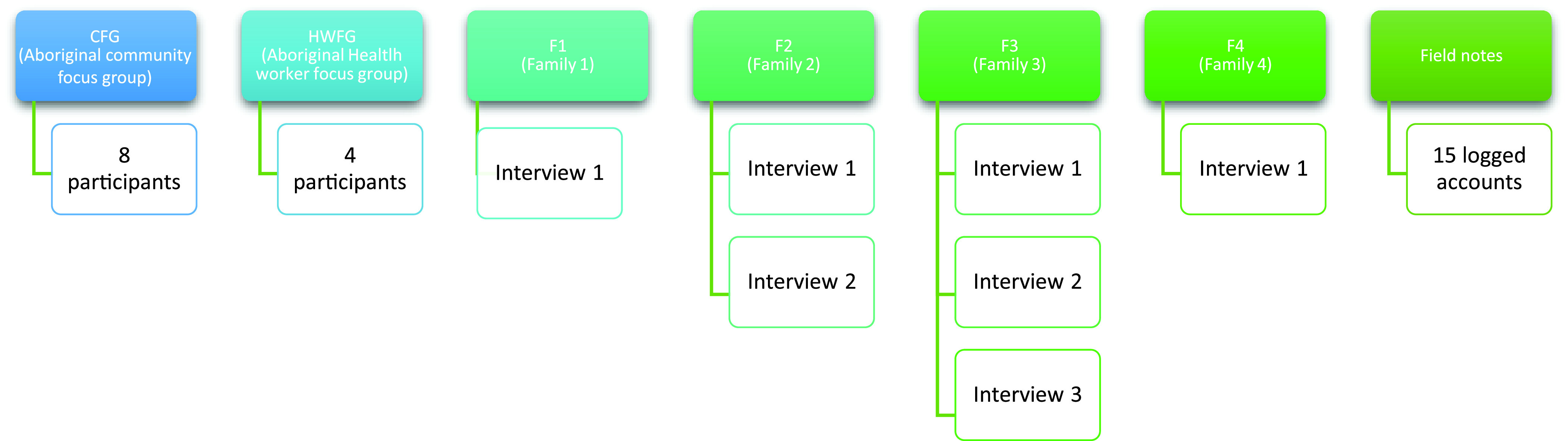
Overview participants and data

A novel qualitative design was employed including photo-elicited interviews (*n* = 7), focus groups (*n* = 2), and field notes (*n* = 15). Photo-elicited interviews (Langhout, 2014) were used as a culturally sensitive way to enable participating families to yarn about their experiences of the Pepi-Pod® program and self-direct their narratives. Seven families agreed to participate in the study and received Pepi-Pods® and Pepi-Pod® education. Of these, four families progressed to interviews. Of those that did not progress, two families moved to regional South Australia and a further family was not contactable. With an aim of conducting three interviews with each remaining family (antenatal, 2 weeks and 12 weeks post-birth), family interviews ranged from one to three; F1 = 1, F2 = 2, F3 = 3, and F4 = 1 (Figure 1).

Interviews were conducted as ‘*research topic yarning*’ (Bessarab and Ng’Andu, 2010) with a community member employed as an Aboriginal Research Liaison Officer (ARLO). Yarning is relational, demanding human to human interaction that enables two way knowing and learning (ibid). This was essential as the innovation was new to both health workers and participating families.

One focus group was held with participating health professionals (*n* = 4), and another with a community group (*n* = 8). Field notes (*n* = 15) provided context for findings and provided opportunities for critical cultural reflection. They comprised handwritten observations, reflections of yarnings, and summaries of team research discussions.

### Ethical considerations

Ethical approvals were received from the South Australian Aboriginal Health Research Ethics Committee (04-16-660), Flinders University, Social and Behavioural Research Ethics Committee (OH-00094), and the Women and Children’s Health Network Human Research Ethics Committee (HREC/16/WCHN/96). Particular attention was given throughout the research to ensure it was conducted in ways that might be experienced as culturally safe. This included consideration of spirit and integrity, reciprocity, respect, equity, cultural continuity, and responsibility (NHMRC 2018).

All authors used the public forum of the RAG to reflexively explore their own cultural positions related to Aboriginal and Torres Strait Islander ways of knowing and being, and the research process. Following Dew et al (2019), this enabled the research team to openly discuss approaches to ensure they were not unwittingly perpetuating colonizing practices and further marginalizing Indigenous peoples within research

### Training, recruitment, and data collection

A collaborative training day was held as a diffusion of innovation strategy (Rogers, 2003) to enable collective learning and to explore how Pepi-Pod® education could be incorporated into existing practices. Further, it enabled connections between participating health workers, clarification of research processes, and identification of strategies for ongoing communication

Family participants were recruited through participant information sheets provided by Aboriginal Maternal Infant Care workers (AMIC) workers in the Aboriginal Family Birthing Program. Participants were Aboriginal families expecting a baby in the Adelaide metropolitan area who were willing to participate in the Pepi-Pod® project. Inclusion criteria included being over 16 years old, having a non-risk pregnancy and no medical complications during pregnancy and birth.

Following receipt of consent to contact the ARLO, the AMIC worker provided participant contact information to the ARLO who made contact with the family. The ARLO then yarned with the family about participation and sought written consent. As evidence of collaboration and respect, recruitment and communication methods changed throughout the research to meet the competing needs of the ACCs, AMIC workers, and ARLO. Each change was approved as a modification by the ethics committees. Despite this continued engagement with industry and community, recruitment was challenging. From a total population pool of 76 families over 10 months, only seven families were recruited (Table 1).

**Table 1.** Recruitment

Total pool	Did not meet inclusion criteria:			Study not discussed due to time/work constraints	Declined/not interested	Agreed
	Out of geographical region	Complex presentation	<16 years			
76	16	14	4	25	10	7

The data collection period was one of extreme pressure as it coincided with a major organizational Model of Care review and an unexpected reduction in the AMIC and ACC workforce. Due to increased workload pressures, AMIC workers indicated that they had reduced capacity to support recruitment and were no longer able to visit the family in their home and deliver the Pepi-Pod® and provide safe sleep education to families as originally planned, or hand over to the ACCs who worked in community with the Child and Family Health Service. The ARLO increased her involvement by delivering the Pepi-Pod® to families, reaffirming and providing the Pepi-Pod® safe sleep education and liaising with the ACCs.

Questions explored with families in yarning episodes included ‘*Please tell me about what is in this photograph?*’, ‘*What made you think of taking this picture?’*, ‘*How does this picture relate to your baby’s sleep?*’, ’*How does this picture relate to your decisions around using the Pepi-Pod®?*’, and ‘*Is there anything else you would like to share with me about the Pepi-Pod®?*’.

**Figure 2.**
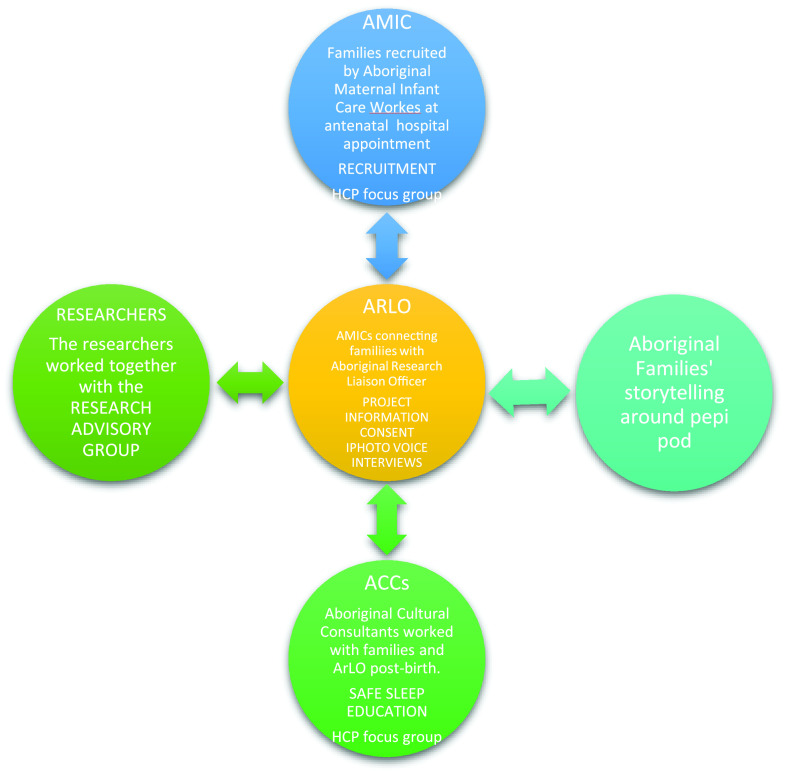
Pathways, process, and research partnerships

The pathway, process, and research partnership between ARLO, AMICs, ACCs, Aboriginal families, and researchers is represented below in Figure 2.

A focus group discussing the Pepi-Pod® program was held with health care professionals (HCPs) who were involved in the program (*n* = 4). They were asked to describe their experiences of working with the Pepi-Pod® program and families engaged in the program. Members of the community focus group (CFG *n* = 8) were recruited by the ARLO. They were female Aboriginal community members who expressed interest in having a say about the Pepi-Pod® program.

### Data analysis

All data were entered into NVivo 11 where it underwent initial descriptive coding (Saldana, 2016) by the first two authors. Descriptive codes were thematically clustered, aggregating all data sets. When codes were clustered, they were renamed using in vivo labels to respect the voices of Aboriginal women who took part in the study. As findings emerged throughout the process of the study, they were cross-checked with the ARLO and RAG for reliability.

We present the themes through short descriptive passages as a narrative strategy to reflect the research voices of Aboriginal community and family members. Using narratives as a way of conveying information is of social importance in transmission of cultural knowledge (Ware et al., 2018). By doing this, we attend to the agreed goal of making the research culturally safe in the presentation of findings. Four themes were identified and are presented below. They include ‘*you don’t have to worry’*, a way of sharing knowledge, ‘*looks like a bread box’* and need for consistent safe sleep messages.

## Findings

### Theme 1: ‘you don’t have to worry’

Keeping Aboriginal babies safe and comfortable was important to participant mothers and the health care professionals connected to the study. F2 for example said, ‘*He was safely, comfortably sleeping. I want my child to be safe and comfortable*’ (F2 1). F3 reported that her baby looked ‘*peaceful and safe*’ in the Pepi-Pod® with F4 describing her baby in ‘*his own little comfort zone’*. As a sleep enabler, participant mothers said for example ‘*it’s a lot easier just to get her to sleep, she is comfortable in in it’* (F1 1).

For participants this meant that ‘*you don’t have to worry. I can see him, I can feel him, I can touch him, I can hear him. You know what I mean?’* (F2 2). Like F2, F3 reported that this lack of worry meant she slept better ‘*you know when you get in that deep sleep and you can feel the Pepi-Pod® next to you’*. Mothers who trialled the Pepi-Pod® program reported a sense of comfort ‘*I know he’s alright and I can watch him right there in front of me’* (F2 1). F2 also found comfort knowing that her baby could see her from the Pepi-Pod® when he woke up. CFG participants liked that there was airflow in the Pepi-Pod®; that they could see through the plastic, and that babies could not ‘*smother their face*’ as they did in bassinettes (CFG).

The safety and protection offered through use of the Pepi-Pod® program was identified by both CFG members and family participants. As a bounded safe space, it provided protection from other children, pets and the ground (CFG, F1-1 F3 1 & 2). F1, for example, said that she places the Pepi-Pod® on the floor next to her bed so that when the ‘*other kids just come and jump on the bed*’ the baby is safe. Similarly, F3’s toddler has learnt ‘*when she’s in there he can’t go near her. That’s her little safe spot*’ (F3 1). *‘He knows he can just have a look at her when she’s in there’* (F3 2). Community women talked of babies being safe on the ground in their Pepi-Pod® during trips out to the clothesline.

The Pepi-Pod® was reported to be used for most sleeps during day and night, with one mother suggesting that she still cuddled her baby to sleep during the day, clarifying that ‘*I’ll lay down with him but I don’t go to sleep*’ (F2). It was used in and beside the family bed, on the couch, on the dining table on the ground outside the house. The majority of mothers reported enjoying the portability of the Pepi-Pod® during the day whereby the baby in the Pepi-Pod® could easily be picked up and moved, for example, to a cooler room in hot weather (F3), or from the loungeroom in the evening to the family bed at night (F2). F3 reported that after the 2–3 am feed she sleeps her baby in the bed ‘*next to me*’ because she wakes up if she goes in the Pepi-Pod®, saying ‘*we’ll put her in there, but then she’ll just cry*’.

Community women suggested that it ‘*would be actually really good to take camping for baby or take away to somebody’s house or sleepovers instead of a cot*’ (CFG). Family participants confirmed this when F2 for example said, ‘*if I go stay anywhere else, I’ll take it with me’*. F1 and F3 also reported taking it for sleepovers or to a babysitter’s house.

### Theme 2: a way of sharing knowledge

Engagement with the Pepi-Pod® program provided a space for intergenerational knowledge transfer. Health care professionals and the families all thought the Pepi-Pod® and the education was ‘*a good idea*’ and ‘*actually very sensible*’ (CFG). Grandmothers explained that although the Pepi-Pod® and its accompanying information was great, the best thing was that it enabled grandmothers and new mums to spend time together. It was a catalyst for young people to learn new parenting skills around safe sleeping and keeping Aboriginal babies safe; increased their levels of cultural knowledge and returned an asset to the community.

Community women found the overrepresentation of SIDs deaths for Aboriginal babies ‘*shocking and quite upsetting*’ (CFG) with one grandmother suggesting ‘*I wish I knew this when I had kids*’ (CFG). They suggested that ‘*all mothers wrap*’ their babies and that babies who also co-sleep ‘*get hot really fast*’. They suggested that education about sleeping babies safely ‘*should start from the hospitals*’ particularly ‘*when they know they’re going home or before they go*’ so that ‘*they’ve got that knowledge*’ (CFG).

### Theme 3: looks like a bread box

A range of reasons were provided for decisions not to use the Pepi-Pod®. F4, for example, wanted to try the Pepi-Pod® but found that she ended up co-sleeping like she did with her other children. F4 said that she ‘*just forgot it was there’*. This was because she put it ‘*under his cot’* due to lack of space, showing the ARLO that neither the cot nor the Pepi-Pod® were being used. F4 thought the ‘*hard plastic*’ stopped it looking ‘*cosy’* or ‘*comfortable’* and that her baby wanted ‘*skin to skin and smell as well’* which she wouldn’t get in the Pepi-Pod® or the cot. Giving the Pepi-Pod® a go ‘*at two to three weeks*’ when she was breastfeeding all the time ‘*she wouldn’t have a bar of it*’. When her baby was 4 months old F3 said she stopped using the Pepi-Pod® ‘*because she keeps hitting her hands when she’s rolling on the plastic and it’s annoying her*’.

To counter these challenges participants made many suggestions for improvement to the design of the Pepi-Pod®, these included practical suggestions such as ‘*non-slip rubber feet on the corners*’ and ‘*handles for carrying*’, and ideas to make the Pepi-Pod® more attractive to young mothers. Through much laughter the women in the CFG said that the pod looked like a ‘*crate’*, ‘*bread box’* or ‘*storage box used for camping’*. They suggested that uptake might be improved if it looked ‘*more attractive’* because ‘*young mothers…the first thing they want to do is make everything for the baby look pretty’* (CFG). They suggested that the Pepi-Pod® the plastic could be tinted, or families could paint the Pepi-Pod® leaving the viewing window clear. If the Pepi-Pod® were an oval shape, it could fit more snuggly into an arm when sleeping ‘*like the long Coolamon type shape*’ (CFG).

### Theme 4: need for consistent safe sleep messages

Much of the community women’s’ conversation was spent exploring how information about safe sleeping has changed over time and is confusing. This extended into concern that messages that mothers receive when they are pregnant is often different to hospital information and then again when they go home. One grandmother said, ‘*now if I’m given a baby my first thought is oh which way am I supposed to do it these days because I’ve been taught every different way*!’ (CFG). Other participants joined in with: we put our babies ‘*on their belly and hands like this’*, ‘*absolutely and put their head to one side’* then ‘*lay them on their side and roll this beside them’*. They agreed that the information provided with the Pepi-Pod® was ‘*great’*. It was clear, easy to understand, and stopped the confusion.

Most of all, the participants thought that safe sleep education is a must for all Aboriginal families because ‘*our babies are dying*’ and education must start before babies are born (CFG). All agreed that keeping babies safe was important, but this did not extend to acceptance of health professionals coming into their homes to check and document where babies are sleeping. ‘*When health care people come in and ask to look where baby is sleeping it’s too controlling and too invasive – it’s like “are you serious*?”’ (CFG). While participants wanted continuity of messages and messaging, they did not believe that *‘invading their privacy’* achieved this (CFG).

An additional factor shaping consistency of messaging in this research were the challenges of service delivery identified by health professional participants. Despite early engagement and commitment to the safe sleep research and messaging (field notes 1, 3, 4, 5, 9, 10), high staff turnover and staff shortages led to increased work pressures where research messaging and engagement was not always consistent (HWFG).

## Discussion

### It is safe, but is it culturally appropriate?

Our research collaboration explored with a small cohort of Aboriginal families, community members, and health professionals, whether the Pepi-Pod® program was culturally appropriate to use in a South Australian metropolitan context. The study identified that participants were in two minds about the intervention, specifically the cultural appropriateness of the Pepi-Pods® themselves and the cultural safety of program delivery.

To be able to determine culturally appropriateness, researchers must be able to understand cultures and subcultures within a population, understand health behaviors according to cultures, and be able to apply this knowledge in planning and execution of research (Tan and Cho, 2019). In this study, we authentically report participant voices telling us their views about the cultural appropriateness of the Pepi-Pod® program. All family members expressed experiencing a comforting level of safety when using the Pepi-Pod®. However, they identified that the Pepi-Pod® itself could be improved to better correlate to Aboriginal culture, knowledge, practices, and symbols, and understanding and respecting the original culture and context. The pods felt foreign and cold, created in plastic in a rectangular form not easily embraced or included into bed. Participants’ dislike of the ‘bread box’ appeared to be rooted in something deeper; a lack of connection to culture. Participants all reflected that if the baby box looked more like a Coolamon, a traditional Aboriginal baby sleeper and carrier, more mothers might find it easier to use.

Cultural safety refers to people feeling able to access and use a service provided by people from another culture without losing their own culture in the process (Ramsden, 2002). Community members and Aboriginal health workers health spoke more about the challenges of providing and receiving culturally safe care than the Pepi-Pod® itself. They specifically referred to problematic surveillance and inconsistent safe sleep messaging. Mainstream health services historically present as a barrier to accessing care (Stanford et al., 2019) for Aboriginal peoples. Surveillance and inconsistencies contribute to these barriers. This small study highlighted that these challenges remain, despite the participating health service leading the way in developing and promoting the culturally specific Aboriginal Family Birthing Service (see e.g., Brown et al., 2017)

The many touchpoints through antenatal care, birthing, postnatal care, and community child health were problematic for families. We posit that fragmentation of service delivery contributed to fragmentation of Pepi-Pod® program safe sleep messaging. This meant that safe sleep education was not always experienced as culturally safe by the families. We cannot extract specific safe sleep data from reported experiences of mainstream service provision because of the natural setting of the research. Concerns remain about continuity of messaging and surveillance for Aboriginal families accessing mainstream health services across the first 1000 days.

The families in this study used the pods, appreciated the program, and liked that it informed and educated them around safe sleeping. We have no data informing us why the remaining eligible families did not take up the program. A larger study that depends less on the research contribution of mainstream health workers would be needed to explore this further.

### Intergenerational knowledge transfer

Families and community members highlighted the positive aspects of intergenerational conversations prompted by the Pepi-Pod® program. Historically First Nations communities follow traditions where older people of a clan or tribe teach children from infancy to adolescence. Despite this practice diminishing due to colonization, assimilation, and segregation (Fox, 2004), it remains important in Aboriginal cultures today, forming the basis of intergenerational knowledge transfer (Singleton et al., 2009). This study opened opportunity for intergenerational knowledge transfer about safe sleep and parenting. This can increase self-worth, promote realization of abilities, and valuing of culture (Singleton et al., 2009). Valuing these relationships is an essential step in creating responsive, culturally respectful, and effective early childhood health care for Aboriginal infants and their families (Harrison et al., 2017). As such the Pepi-Pod® program provided a platform for increased culturally safe care in a mainstream health service.

The colonization of Australia brought significant change and interruption on the lifeways of Aboriginal and Torres Strait Islander peoples, including forced removals onto missions and reserves (Griffiths et al., 2016). The legacy of dispossession is ongoing socioeconomic disadvantage and racial discrimination (Paradies et al., 2015). Indigenous peoples have survived and thrived, grounded by relationship to country, family, and culture. Provision of culturally appropriate safe sleep support and service provision for Aboriginal infants and their families must be grounded in the strengths emanating from Aboriginal cultural lifeways. Embedding elements of culture into Aboriginal maternal and child health care is important for successful health outcomes. Bertilone et al. (2017), for example, found that employing Aboriginal grandmothers in maternity services enabled intergenerational knowledge sharing and spiritual and cultural guidance. Incorporating intergenerational strategies such as this may strengthen the Pepi-Pod® program for future use in mainstream health services.

### Challenges and strengths

In this study, the research collaboration included frontline Aboriginal and non-Aboriginal health workers, industry leaders, services providers, and researchers. Despite this, engagement with the innovation was low. Challenges of the natural setting included workload time pressures, organizational change, and a reliance on health workers who are not researchers. Capacity for collaborative research was further limited by different internal operational systems governing health worker activities. Health services have a responsibility to shift from simply providing safe sleep information to enabling safe infant sleep in action (Young et al., 2014). Enacting this will remain elusive until links between culture, practice, and research are explicitly supported.

While the cohort for this study was small with 16 participants overall, 7 interviews, 2 focus groups (FG1 = 4 participants, FG2 = 8 participants), and 15 field notes, it remains relevant given the cultural focus of the study. Guest, Bunce and Johnson (2006) found that saturation was reached at 6 to 12 interviews. The benefits of undertaking a small, local study enabled clear identification of process successes and limitations to inform scaling in knowledge translation.

## Conclusion

The findings suggest that families, community members, and health care professionals believe that the Pepi-Pod® program may enrich Aboriginal families’ lives evoking feelings of comfort and safety. If the design of the pods was improved to fit more easily in a bed or in an embrace, such as a Coolamon uptake may improve. Opportunities for intergenerational knowledge transfer were identified as positive throughout the Pepi-Pod® program offering a culturally appropriate way forward for health service designers.

Confusion around safe sleep messaging existed with a call for streamlining of safe sleep education for families. This extended to streamlining the service touchpoints across the first 1000 days for Aboriginal families accessing mainstream health services. Mainstream health services have a responsibility to ensure that all practices are experienced as culturally safe (The Wardliparingga Aboriginal Research Unit of the South Australian Health and Medical Research Institute, 2017). This research collaboration is evidence that South Australian health services are working with community and researchers towards this goal and are committed to translating research findings into practice.
