# “It looks like a breadbox”: a pilot study investigating implementation of the Pepi-Pod® program with Aboriginal families in metropolitan South Australia – CORRIGENDUM

**DOI:** 10.1017/S1463423621000803

**Published:** 2022-02-18

**Authors:** Julian Grant, Nina Sivertsen, Janiene Deverix, Alice Steeb

The first sentence of the second paragraph is incorrect. It should instead read as follows:

In South Australia (SA), there were 27 deaths of Aboriginal infants between 2005 and 2014 attributed to SUDI, which often occurs after an infant is placed to sleep (Child Death and Serious Injury Review Committee 2014).

The Authors would like to apologise for this mistake.
